# Supplementary material for: Active control of anapole states by structuring the phase-change alloy Ge2Sb2Te5
Source: Nat Commun. 2019 Jan 23;10:396. doi: 10.1038/s41467-018-08057-1 (PMC6344509; doi:10.1038/s41467-018-08057-1)
Supplement: Supplementary file 1 — Supplementary Information [file 41467_2018_8057_MOESM1_ESM.pdf]

Supplementary Information:

Active control of anapole states by structuring the phase-change alloy $Ge_2Sb_2Te_5$

Jingyi Tian[1,2,†], Hao Luo[1,†], Yuanqing Yang[3,*], Fei Ding[3], Yurui Qu[1,4], Ding Zhao[5], Min Qiu[1,6,7,*], and Sergey I. Bozhevolnyi[3]

[1]*State Key Laboratory of Modern Optical Instrumentation, College of Optical Science and Engineering, Zhejiang University, Hangzhou 310027, China*

[2]*Department of Applied Physics, Royal Institute of Technology, KTH, 10691 Stockholm, Sweden*

[3]*SDU Nano Optics, University of Southern Denmark, Campusvej 55, DK-5230 Odense, Denmark*

[4]*Department of Physics, Massachusetts Institute of Technology, Cambridge, MA 02139, USA*

[5]*DTU Danchip/Cen, Technical University of Denmark, Kongens Lyngby 2800, Denmark*

[6]*School of Engineering, Westlake University, 18 Shilongshan Road, Hangzhou 310024, China*

[7]*Institute of Advanced Technology, Westlake Institute for Advanced Study, 18 Shilongshan Road, Hangzhou 310024, China*

E-mail: yy@mci.sdu.dk; minqiu@zju.edu.cn

[†]These authors contributed equally to this work.

[*]To whom correspondence should be addressed.

Supplementary Note 1: Optical constants of the GST material

The refractive indices of the amorphous (aGST) and the crystalline GST (cGST) thin films were experimentally determined by a spectrophotometric approach [1]. For intermediate phases of the GST material, their optical constants were estimated by using the Lorentz–Lorenz relation [2] as described in the Methods section.

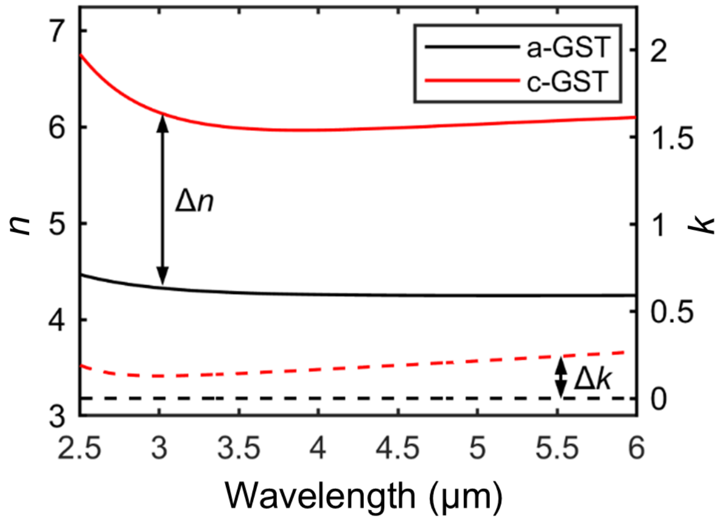

Supplementary Figure 1. Refractive indices of the amorphous (aGST) and the crystalline GST (cGST), produced by magnetron sputtering deposition.

Supplementary Note 2: Multipole expansion for GST spheres with progressive crystallinities and the effects of loss

To examine the physical origins of the dynamic scattering bright and dark states in the GST spheres, in Supplementary Fig. 2**a** we plot the multipole expansion of the scattering response of the GST sphere with varied crystallinities. It is observed that the progressive shifting between the scattering maxima and minima is indeed attributed to the excitation of the ED and the anapole states. Supplementary Fig. 2**b** provides the total electric field distribution of three representative scattering states (MD, ED, and anapole) as supplements to Fig. 1**d**–**f** in the main text.

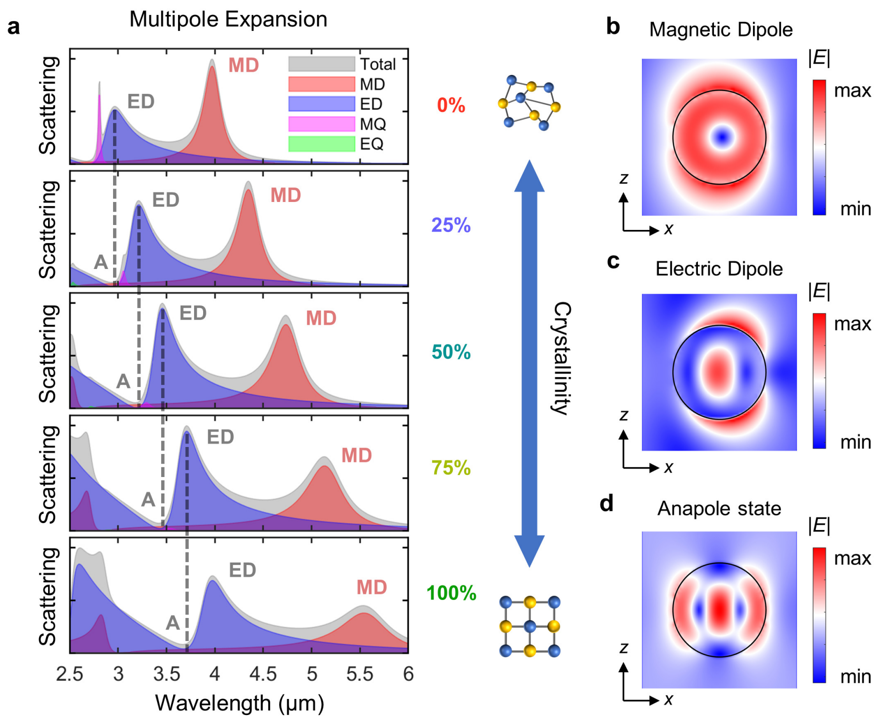

Supplementary Figure 2. (**a**) Progressive multipole decomposition of the scattering spectra of the GST sphere in Fig. 1. (**b**) Total electric field distributions of the three representative scattering states, i.e. MD, ED, and the anapole state.

Note that the GST material is lossy in the mid-infrared range, here we also analyze the effects of loss on different Mie states. As shown in Supplementary Fig. 2**a**, the partial scattering of ED features an evident Fano line shape due to the interference between a resonant eigenmode (internal) and non-resonant background pathway (external) [3]. To extract the Q-factor, the partial scattering contribution of ED at the anapole states was fitted into a Fano line shape given by [4]:

$$\sigma_{\mathrm{ED}}(\omega) \propto \frac{(q\frac{\Gamma}{2} + \omega - \omega_0)^2}{(\frac{\Gamma}{2})^2 + (\omega - \omega_0)^2} \qquad (1)$$

with ω_0 is the central resonant frequency; Γ is the full-width at half-maximum (FWHM) of the resonance; q is the asymmetry parameter describing the ratio between

the resonant scattering and the non-resonant background. An excellent agreement between the Mie calculation and the Fano fit can be seen in Supplementary Fig. 3**a**. For all the crystallinities, the asymmetry parameter q is close to 1, indicating the resonant and the non-resonant pathways have similar amplitudes. The Q-factor was then determined by $Q = \omega_0/\Gamma$, as plotted in Supplementary Fig. 3**b**. We can then quantitatively conclude that the ED scattering does not exhibit dramatic broadening linewidths with increasing crystallinities. This is because a larger crystallinity in GST would bring in increases in both real and imaginary parts of the refractive index $n = n_0 + ik$. For the ED resonance, a larger n_0 would make the structure a more perfect scatterer with a smaller radiative damping and thus lead to a higher Q-factor [5]. Meanwhile, a larger k would result in larger dissipative damping with a smaller Q-factor. Such a trade-off explains why all the ED resonances in Figs. 1, 3 and 4 do not show substantial linewidth broadening and also accounts for the appearance of the maximized Q-factor at $C = 50\%$ in Supplementary Fig. 3**b**.

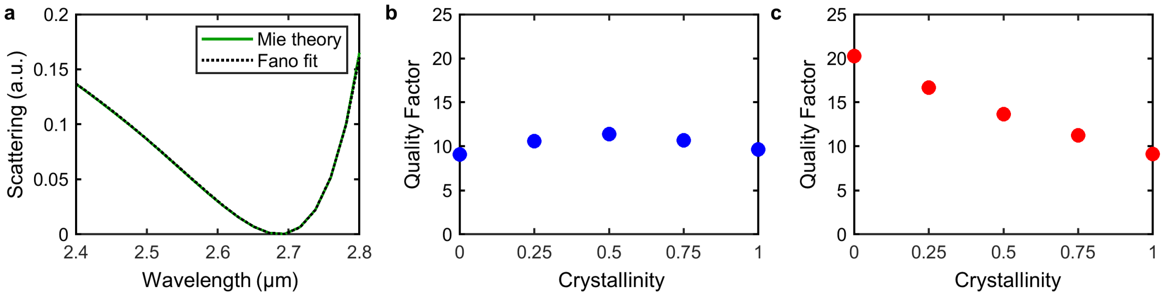

Supplementary Figure 3. (**a**) Fano fitting of the asymmetric line shape of ED contribution at the anapole state in aGST nanosphere. (**b, c**) Relation between the Q-factor of the anapole states (**b**) and the MD state (**c**) with respect to different crystallinities.

In contrast to the asymmetric Fano line shape of the ED contribution, the MD response manifests a symmetric Lorentzian line shape. This is because the internal resonance arising from the circular displacement currents at the MD state is much stronger than the background pathway, thereby dominating in the interference with $q \gg 1$ in Eq. (1). Thus, we can directly obtain the FWHM from the spectra and determine the Q-factor of the MD resonances, as shown in Supplementary Fig. 3**c**. A clear decline in the Q-factor with increasing crystallinities could be seen. We attribute this response to the strong resonant feature at MD states and its large field concentration inside the particle. As such, the increase in k would have a much larger impact on the linewidth than the increase in n_0, which significantly decreases the Q-factor.

Supplementary Note 3: tunable scattering directionality of GST spheres

The broadband active tuning of the Mie resonances in the structured GST can lead to a variety of interesting phenomena. Here we exemplify this point by considering the same GST nanosphere ($R = 450$ nm) as in Fig. 1. The scattering spectra of the GST sphere with three different crystallinities C are plotted in Supplementary Fig. 4**a**, showing the investigated wavelength λ_c at 3.97 µm with a dotted line. To depict the far-field scattering patterns, the same coordinate as in Fig. 1 is adopted, in which the incident wave propagates along the x-axis with the polarization of the electric field along the z-axis.

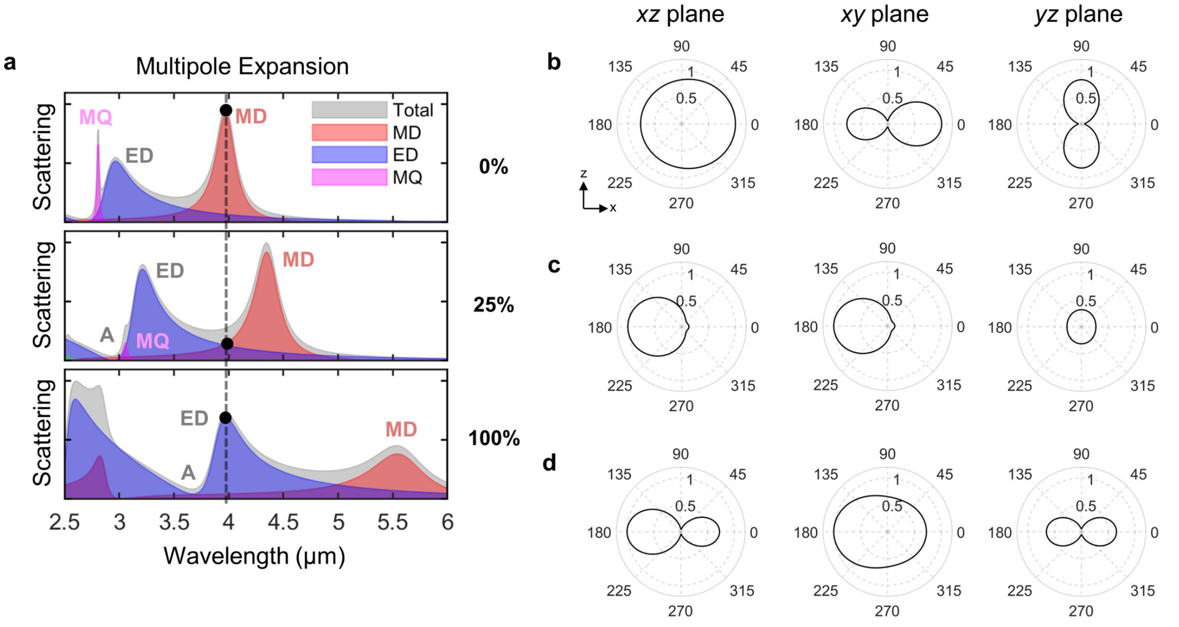

Supplementary Figure 4. (**a**) Progressive multipole decomposition of the scattering spectra of the GST sphere in Fig. 1. (**b-d**) Far-field scattering patterns of the GST sphere at $\lambda_c =$ 3.97 µm with three different crystallinities, i.e. $C = 0\%$, 25%, and 100%.

When the GST sphere is at the amorphous state, it supports a magnetic dipole resonance at λ_c. The far-field scattering in Supplementary Fig. 4**b** shows a typical radiation pattern of a magnetic dipole oriented along the y-axis. By contrast, after introducing a moderate phase change of 25%, the scattering spectrum shows an intersection between the electric and magnetic dipole contributions. The spectral overlap and equal far-field strengths of the two dipoles indicate the satisfaction of the second Kerker condition [6], as confirmed by the unidirectional scattering in the backward direction. When the phase change continues increasing, the sphere finally reaches its crystalline state with its scattering similar to that of a typical electric dipole oriented along the z axis, i.e., the scattering pattern (Supplementary Fig. 4**d**) transforms in orthogonal to that of the amorphous sphere. Therefore, mode shifting between magnetic and electric dipole resonances could also be realized with the GST sphere, which may make a fundamental impact on many intriguing physical phenomena related to Mie resonances.

Supplementary Note 4: scattering efficiencies Q_{scat} of the aGST and the 25%-cGST spheres

To verify the "nearly–dispersionless" behavior of the switching effect, in Fig. 2c we plot the scattering contrast of the GST spheres with two different crystallinities, i.e. $C = 0\%$ and $C = 25\%$ and then analytically investigate the conditions for a rigorous switching (Fig. 2d). Here, as supplements, the scattering efficiencies Q_{scat} of the aGST and the 25%-cGST spheres are provided in Supplementary Fig. 5**a, b**, respectively.

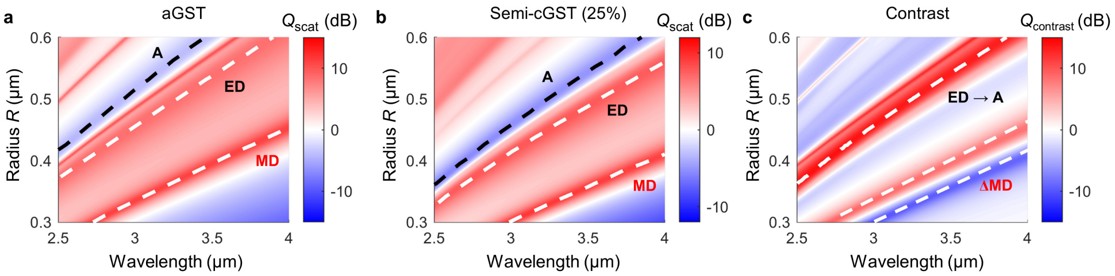

Supplementary Figure 5. Scattering efficiencies Q_{scat} of amorphous (**a**) GST spheres and 25%-cGST (**b**) with varying radii R. (**c**) The scattering cross sectional contrast of GST spheres at the two phases, which is defined as $Q_{\text{contrast}} = Q_{\text{scat-aGST}}/Q_{\text{scat-25\%-cGST}}$. We mention that the Supplementary Fig. 5**c** is the same as Fig. 2c and it is provided here just for ease of reference.

Supplementary Note 5: AFM measurement of the disks' geometric profiles

The geometric profiles of the fabricated GST nanodisks were determined by AFM, as shown in Supplementary Fig. 6.

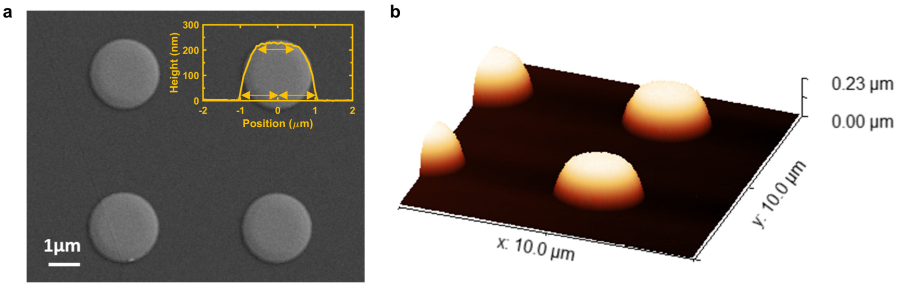

Supplementary Figure 6. Geometric profiles of the fabricated GST nanodisks. (**a**) A zoom-in SEM image as a supplement to Figure 3a in the main text. The inset shows the AFM data of the cross section of the GST disk. The ratio between the bottom and the top radius was measured as 2, the same as devised in preliminary numerical designs. (**b**) 3D AFM data of a fabricated array of GST disks.

We also note that the phase change of the GST material may introduce a volume reduction of $\sim 6\%$ from the amorphous to the crystalline state in thin film [7]. However, it is not clear how such a change would behave and evolve in 3D GST nanostructures, i.e., whether it occurs homogenously along all the directions and linearly in time or not. In our study, we observed a 5% height reduction between amorphous and crystalline GST nanodisks without any noticeable changes in their lateral sizes or surface topology (Supplementary Fig. 7**a**). Given the large diameter-to-height ratio of the disks in our study, such a subtle change would not influence the spectral position or the strengths of the resonances (Supplementary Fig. 7**b**).

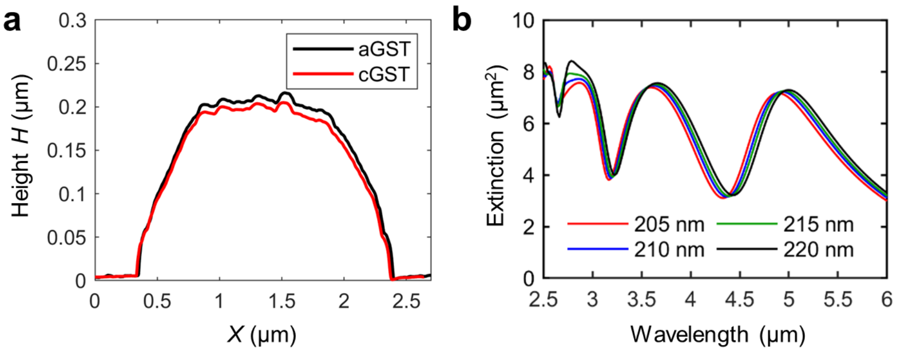

Supplementary Figure 7. (**a**) The AFM scans at the center of the GST disk at the amorphous (black) and crystalline (red) states. (**b**) Simulated extinction spectra of a cGST nanodisk with $D = 2\,\mu\mathrm{m}$ and different heights H.

Supplementary Note 6: influences of the pitch size, the absorption loss, and the substrate on the scattering states

In this supplementary section, we discuss the impacts of the pitch size (inter-particle distance), the absorption loss of the GST material, and the existence of the substrate on the investigated scattering states and multipolar responses in GST nanodisks.

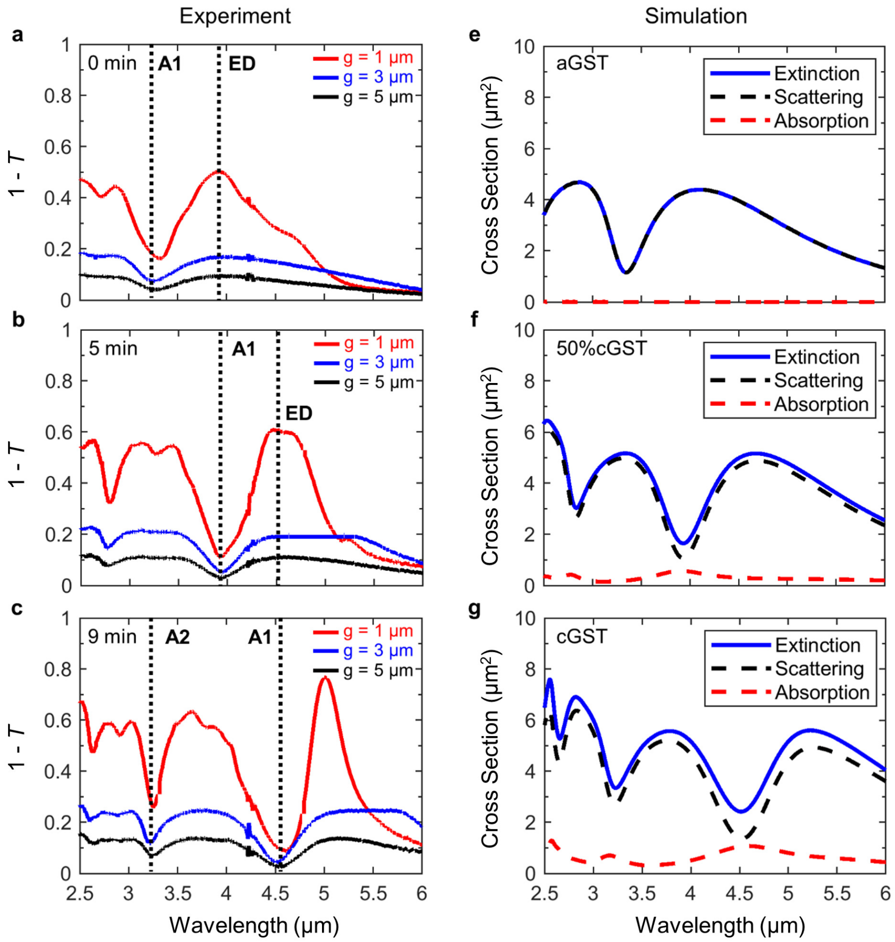

Supplementary Figure 8. (**a**–**c**) Extinction spectra of the GST disks with a bottom radius $R = 1\,\mu\text{m}$ and different pitch sizes g. (**d**–**f**) Extinction, scattering and absorption cross section spectra of individual GST disks ($R = 1\,\mu\text{m}$) at three different crystalline phases. The influence of the absorption is mild in all cases and the extinction features (pronounced peaks and dips) correlates well with those on the scattering spectra.

As shown in Supplementary Fig. 8**a**–**c**, for different pitch sizes g, the optical coupling between adjacent GST disks does not strongly influence the spectral positions of the investigated ED and anapole states. In particular, there is generally no difference between the case of $g = 3\,\mu\text{m}$ and $g = 5\,\mu\text{m}$. In the main text, to highlight the resonances of the disks and to mitigate the influence of the CO_2 absorption (around $4.3\,\mu\text{m}$), we used the spectra with $g = 3\,\mu\text{m}$.

In Supplementary Fig. 8**d**–**f**, we provide the scattering, the absorption, and the extinction spectra of individual GST disks with different crystallinities. Indeed, as the

crystallinity increases, the absorption of the GST material increases. However, given the relatively low loss of the GST material in the wavelength range of interest, the influence of the absorption is mild (e.g. contribute to $\sim$ 16% extinction at the ED resonance for cGST) and the extinction features (pronounced peaks and dips) correlate well with those on the scattering spectra.

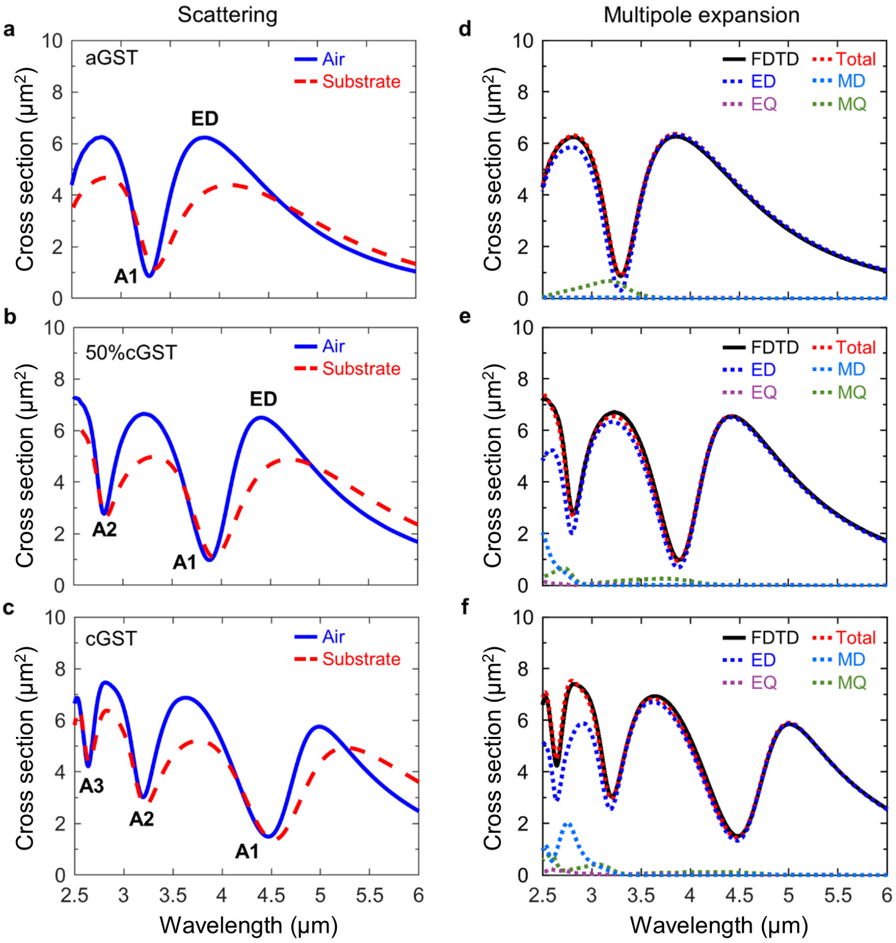

Supplementary Figure 9. (**a**–**c**) Simulated scattering spectra of individual GST nanodisks in the vacuum and on the substrate. (**d**–**f**) Multipole expansion of the simulated scattering spectra. Both fundamental and higher-order multipoles can be clearly identified.

In Supplementary Fig. 9**a**–**c**, we compare the scattering spectra with and without the substrate. Based on the Mie theory [8], the strengths and the spectral positions of the multipolar responses are directly related to the index contrast between the dielectric material and the environment. Since the GST material possesses extremely high indices ($n_{\mathrm{aGST}} > 4$, $n_{\mathrm{cGST}} > 6$), the existence of the substrate ($n_{\mathrm{CaF_2}} \sim 1.4$) thereby only has a very limited impact on the spectral positions of the scattering spectra. The associated multipole expansion in the vacuum (Supplementary Fig. 9**d**–**f**) thus can be applied to identify the ED and the anapole modes in the experiment. It is also worth noting that our multipole expansion approach (see Methods) allows us to unambiguously identify multipolar contributions up to arbitrarily high order.

Supplementary Note 7: Tunable beam steering of a GST metasurface

In addition to the active control of anapole states and presented optical switch in the main text, here we numerically demonstrate an alternative application of GST metasurfaces composed of disk arrays for tunable and efficient beam steering.

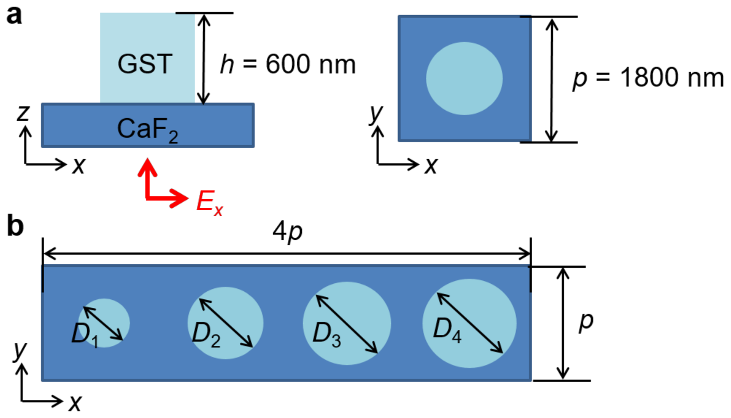

Supplementary Figure 10. (**a**) The cross sections of a unit cell in the tunable metasurface consisting of GST disks. (**b**) Illustration of a supercell consisting of 4 GST disks with varied diameter. To realize the demonstrated functionality, here the diameters D_1 to D_4 are 770 nm, 1130 nm, 1310 nm, and 1340 nm, respectively.

Following the general principle for designing gradient metasurfaces [9], here we first set the height of the GST disks to 600 nm and the period of a unit cell to 1800 nm. By varying the diameter of the disks, the Mie resonances supported by the disks would undergo spectral shifts and thereby exhibit varied phase response at the design wavelength $\lambda_d = 4\,\mu m$. In this way, we can introduce a linear phase gradient along the x-direction, parallel to the incident polarization, as seen in Supplementary Fig. 10. According to the generalized Snell's law [10], the incident light would be anomalously transmitted into a specific angle, as shown in the Supplementary Fig. 11**a**. 87.6% of the transmission is propagating along the +1 diffraction order at λ_d with light in other diffraction orders being strongly suppressed. By contrast, when we introduce a 30% phase change in the GST disks, the refractive index of GST would increase and lead to dramatic redshifts of the supported resonances, thereby limiting the phase variation at λ_d. As such, the GST disk arrays would support a nearly constant phase response along the interface, resulting in the metasurface exhibiting the conventional (zero-order) transmission (Supplementary Fig. 11**b**) with most of the light (95.4%) propagating normally. Hence, in this manner, one can realize a GST metasurface with tunable beam steering by utilizing a simple configuration of GST disks.

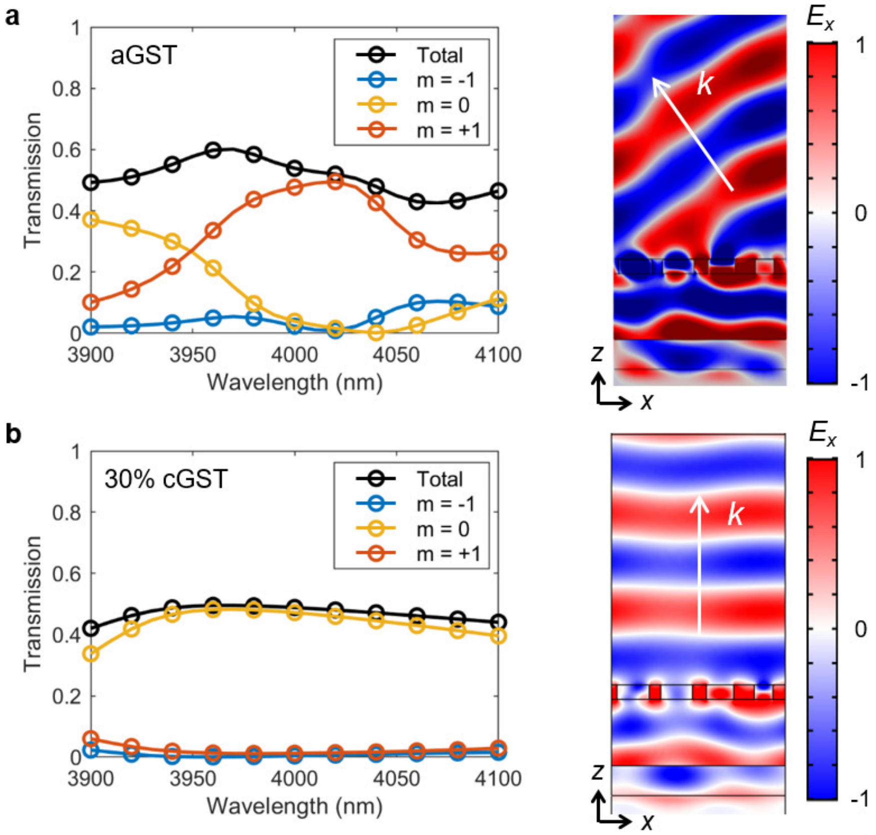

Supplementary Figure 11. (**a**) Transmission spectra (left) for different diffraction orders ($|m| \leqslant 1$) of the GST metasurface composed of amorphous GST disks. The calculated electric field distribution (right) at $\lambda_\mathrm{d} = 4\,\mu\mathrm{m}$ showing anomalous transmission. (**b**) Transmission spectra (left) for different diffraction orders ($|m| \leqslant 1$) of the GST metasurface composed of 30% cGST disks. The calculated electric field distribution (right) at $\lambda_\mathrm{d} = 4\,\mu\mathrm{m}$ showing ordinary (zero-order) transmission along the $+z$ direction.

Supplementary References

1. Du, K.-K., Li, Q., Lyu, Y.-B., Ding, J.-C., Lu, Y., Cheng, Z.-Y. & Qiu, M. Control over emissivity of zero-static-power thermal emitters based on phase-changing material GST. *Light Sci. Appl.* **6,** e16194 (2017).

2. Aspnes, D. Local-field effects and effective-medium theory: a microscopic perspective. *Am. J. Phys.* **50,** 704–709 (1982).

3. Limonov, M. F., Rybin, M. V., Poddubny, A. N. & Kivshar, Y. S. Fano resonances in photonics. *Nat. Photonics* **11,** 543–554 (2017).

4. Fan, P., Yu, Z., Fan, S. & Brongersma, M. L. Optical Fano resonance of an individual semiconductor nanostructure. *Nat. Mater.* **13,** 471–475 (2014).

5. Tribelsky, M. I. & Miroshnichenko, A. E. Giant in-particle field concentration and Fano resonances at light scattering by high-refractive-index particles. *Phys. Rev. A* **93,** 053837 (2016).

6. Sikdar, D., Cheng, W. & Premaratne, M. Optically resonant magneto-electric cubic nanoantennas for ultra-directional light scattering. *J. Appl. Phys.* **117,** 083101 (2015).

7. Weidenhof, V., Friedrich, I., Ziegler, S. & Wuttig, M. Atomic force microscopy study of laser induced phase transitions in $Ge_2Sb_2Te_5$. *J. Appl. Phys.* **86,** 5879 (1999).

8. Bohren, C. F. & Huffman, D. R. *Absorption and scattering of light by small particles* (John Wiley & Sons, 2008).

9. Ding, F., Yang, Y., Deshpande, R. A. & Bozhevolnyi, S. I. A review of gap-surface plasmon metasurfaces: fundamentals and applications. *Nanophotonics* **7,** 1129–1156 (2018).

10. Yu, N., Genevet, P., Kats, M. A., Aieta, F., Tetienne, J.-P., Capasso, F. & Gaburro, Z. Light propagation with phase discontinuities: generalized laws of reflection and refraction. *Science* **334,** 333–337 (2011).
